# Impact of Fe(III) (Oxyhydr)oxides Mineralogy on Iron Solubilization and Associated Microbial Communities

**DOI:** 10.3389/fmicb.2020.571244

**Published:** 2020-11-20

**Authors:** Fengfeng Zhang, Fabienne Battaglia-Brunet, Jennifer Hellal, Catherine Joulian, Pascale Gautret, Mikael Motelica-Heino

**Affiliations:** ^1^Univ. Orléans, CNRS, BRGM, ISTO, UMR 7327, Orléans, France; ^2^BRGM, Orléans, France

**Keywords:** iron-reducing bacteria, *Shewanella*, *Geobacter*, iron (oxyhydr)oxides, solubilization

## Abstract

Iron-reducing bacteria (IRB) are strongly involved in Fe cycling in surface environments. Transformation of Fe and associated trace elements is strongly linked to the reactivity of various iron minerals. Mechanisms of Fe (oxyhydr)oxides bio-reduction have been mostly elucidated with pure bacterial strains belonging to *Geobacter* or *Shewanella* genera, whereas studies involving mixed IRB populations remain scarce. The present study aimed to evaluate the iron reducing rates of IRB enriched consortia originating from complex environmental samples, when grown in presence of Fe (oxyhydr)oxides of different mineralogy. The abundances of *Geobacter* and *Shewanella* were assessed in order to acquire knowledge about the abundance of these two genera in relation to the effects of mixed IRB populations on kinetic control of mineralogical Fe (oxyhydr)oxides reductive dissolution. Laboratory experiments were carried out with two freshly synthetized Fe (oxyhydr)oxides presenting contrasting specific surfaces, and two defined Fe-oxides, i.e., goethite and hematite. Three IRB consortia were enriched from environmental samples from a riverbank subjected to cyclic redox oscillations related to flooding periods (Decize, France): an unsaturated surface soil, a flooded surface soil and an aquatic sediment, with a mixture of organic compounds provided as electron donors. The consortia could all reduce iron-nitrilotriacetate acid (Fe(III)-NTA) in 1–2 days. When grown on Fe (oxyhydr)oxides, Fe solubilization rates decreased as follows: fresh Fe (oxyhydr)oxides > goethite > hematite. Based on a bacterial *rrs* gene fingerprinting approach (CE-SSCP), bacterial community structure in presence of Fe(III)-minerals was similar to those of the site sample communities from which they originated but differed from that of the Fe(III)-NTA enrichments. *Shewanella* was more abundant than *Geobacter* in all cultures. Its abundance was higher in presence of the most efficiently reduced Fe (oxyhydr)oxide than with other Fe(III)-minerals. *Geobacter* as a proportion of the total community was highest in the presence of the least easily solubilized Fe (oxyhydr)oxides. This study highlights the influence of Fe mineralogy on the abundance of *Geobacter* and *Shewanella* in relation to Fe bio-reduction kinetics in presence of a complex mixture of electron donors.

## Introduction

Fe (oxyhydr)oxides are ubiquitous components in several compartments of the critical zone (e.g., soils, sediments, and aquifers) and are present in many different mineralogical forms. Understanding biogeochemical behavior and Fe cycling is fundamental for many scientific communities (Bonneville et al., 2004; Roden et al., 2004). Indeed, the mobility of associated trace elements (TE) is partly controlled by Fe speciation, mineralogy and reactivity (Cornell and Schwertmann, 2003). The natural solubility of crystalline Fe (oxyhydr)oxides is low. However, the interaction with microbes and organic substances can enhance the formation of soluble Fe(III) and increase the availability of Fe and associated TE (Colombo et al., 2014). Many biogeochemical aspects of Fe cycling, including the major microbially mediated and abiotic reactions, have been extensively covered (Melton et al., 2014), together with Fe redox transformations and availability of TE (Zhang et al., 2012), as well as Fe redox cycling in bacteriogenic Fe oxide-rich sediments (Gault et al., 2011). In aerobic environments at circumneutral pH conditions, Fe is generally relatively stable and highly insoluble in the form of (oxyhydr)oxides (e.g., Fe(OH)_3_, FeOOH, Fe_2_O_3_). However, in anaerobic conditions these minerals can be reductively dissolved (Roden and Wetzel, 2002; Roden et al., 2004) by microbial and abiotic pathways (Bonneville et al., 2004; Hansel et al., 2004; Thompson et al., 2006; Shi et al., 2016). In particular, reductive dissolution of Fe (oxyhydr)oxides can be driven by dissimilatory iron-reducing bacteria (DIRB), significantly contributing to biogeochemical cycling of Fe and subsequent TE mobilization (Cooper et al., 2006; Ghorbanzadeh et al., 2017; Levar et al., 2017). Microbial dissimilatory iron reduction (DIR) is a ubiquitous biogeochemical process in suboxic environments (Lovley, 2000; Crosby et al., 2005; Wilkins et al., 2006; Schilling et al., 2019). DIRB use Fe (oxyhydr)oxides as electron acceptors instead of oxygen for oxidizing organic matter. Moreover, the rate of Fe(III) reduction will also influence mobility of TE initially immobilized on or in Fe (oxyhydr)oxides through adsorption or co-precipitation. Crystallinity, specific surface area and size among other factors may influence reactivity of Fe (oxyhydr)oxides in relation to the metabolic activity and diversity of DIRB (Cutting et al., 2009; Aino et al., 2018).

The role of iron-reducing bacteria (IRB) in Fe redox transformations has been evidenced for more than three decades (Lovley et al., 1987; Lovley, 1991; Stern et al., 2018; Meile and Scheibe, 2019; Su et al., 2020), during which more than 100 distinct IRB species have been found. However, *Geobacter* and *Shewanella* are the two most studied IRB genera up to now (Li et al., 2012; Han et al., 2018; Engel et al., 2019; Jiang et al., 2020). Some studies have focused on the observation of secondary mineral formation in presence of *Geobacter* or *Shewanella* strains during the bio-transformation of amorphous, poorly crystalline and highly crystalline iron (oxyhydr)oxides i.e., ferric (ferrihydrite, goethite, hematite, lepidocrocite), ferrous (siderite, vivianite), and mixed valence (magnetite, green rust) (Fredrickson et al., 1998; Zachara et al., 2002; Han et al., 2018). Moreover, molecular mechanisms that occur during Fe reduction have been characterized by electro microbiology for *Geobacter* and *Shewanella* (Nealson, 2017; Shi et al., 2019). Additionally, IRB communities may be influenced by initial Fe mineralogy and the nature of available electron donors. Lentini et al. (2012) compared IRB cultures obtained with different organic substrates, i.e., glucose, lactate and acetate, and different Fe(III)-minerals, i.e., ferrihydrite, goethite and hematite. Type of electron donor was the most important factor influencing community structure, that also varied with the nature of the Fe(III)-mineral. The availability of carbon sources, other than acetate, induced the development of sulfate-reducing bacteria, that could indirectly dissolve Fe(III)-minerals through the production of H_2_S, whereas acetate alone induced the dissolution of ferrihydrite and the development of *Geobacter*. Hori et al. (2015) obtained IRB enrichments from diverse environments with only acetate that favored the selection and isolation of organisms belonging to the *Geobacter* genus. Acetate is a common small organic acid that cannot support fermentation, thus its consumption is generally linked to respiratory mechanisms. However, mixtures of organic substrates can be found in soils and sediments. In order to obtain complementary information on complex IRB communities that could be helpful to make the link with previous experiments involving pure strains only, the present study was performed with a mixture of simple (acetate, formate, lactate, glucose) and complex (peptone) electron donors and focused on the abundance of the two model genera, *Geobacter* and *Shewanella*, in bacterial communities originating from natural environments. Bio-reduction of four different Fe (oxyhydr)oxides presenting contrasting specific surfaces, crystallinity and solubility features, i.e., two freshly synthetized Fe (oxyhydr)oxides, and the two defined Fe-oxides goethite and hematite, was investigated with the obtained IRB enrichments. The objective of this experiment was to assess (1) the dissolution rate of these minerals in presence of mixed IRB communities while inhibiting sulfate reduction, and (2) the influence of the type of Fe(III)-mineral on the relative abundances of *Shewanella* and *Geobacter*, when a complex mixture of organic substrates is provided.

## Materials and Methods

### Soil and Sediment Sampling and Enrichment of Iron-Reducing Bacteria (IRB)

Soils and riverbanks periodically subjected to flooding and thus to cyclic redox oscillations represent one of the surface environments where IRB should actively contribute to Fe bio-reduction. This study was based on IRB enrichments from soil and sediment samples from a riverbank, in a site already studied in terms of Fe and TE total concentration profiles in sediment cores (Dhivert et al., 2015). The sampling site is located in a Loire river channel, in Decize, France (Dhivert et al., 2015). Three samples were collected using an auger and stored under a N_2_ atmosphere: soil from the riverbank (10–15 cm depth), soil from flooded ground (0–7 cm depth) and under-water sediment (7–17 cm depth), which were named D1, D2, and D3, respectively. In order to obtain cultures enriched in Fe(III)-reducing bacteria, enrichment medium containing Fe(III) as electron acceptor and Na-molybdate to inhibit the development of sulfate-reducing bacteria was used. 10 g of each soil sample were inoculated into 200 mL basic medium (composition detailed in the Supplementary Figure S1, Lovley, 2006; Huguet, 2009) autoclaved (121°C, 20 min) then flushed with sterile N_2_ just after autoclaving. The headspace of vials (small volume because 200 mL bottles were used) was N_2_. The following components were added to this medium: 10 mM of Fe(III) Nitrilotriacetic Acid as electron acceptor, 1.5 g L^–1^ peptone, 10 mM of acetate, lactate, and formate, 2 mM glucose as electron donors (Lovley et al., 1989; Coates et al., 1996; Shelobolina et al., 2007; Kwon et al., 2016) in anaerobic conditions, and 0.4 mM of sodium molybdate. Fe(III)-NTA (100 mM stock solution) was prepared by dissolving 1.64 g of NaHCO_3_ in 80 ml water, adding 2.56 g C_6_H_6_NO_6_Na_3_ and 2.7 g FeCl_3_⋅6H_2_O, bringing the solution up to 100 ml, flushing with N_2_ and filter sterilizing (0.2 μm, Millex-GP Syringe Filter, 33 mm diameter) into a sterile, anaerobic serum bottle. Sterilization of the electron donors was performed by autoclaving for acetate, lactate and formate, and filtration at 0.2 μm for peptone and glucose. Sodium molybdate was autoclaved. All stock solutions were kept anaerobic under N_2_ after sterilization. Cultures were incubated at 20°C under agitation (100 rpm) for 10 days. Samples (1.5–2 mL) were collected in an anaerobic glove box, filtered at 0.45 μm (Millex -GP Syringe Filter, 33 mm diameter) and analyzed for Fe(II) content in order to evaluate Fe(III) reduction. After 3–5 steps of sub-culturing (inoculation at 10% in fresh medium, every 2 weeks), the three Fe-reducing cultures were able to reduce 10 mM Fe(III) into 1–2 days, and were used as inocula for the following IRB incubation experiments (see section “IRB Incubation Experiments”).

### Fe(III) (Oxyhydr)oxides

Two fresh Fe (oxyhydr)oxides were synthesized in the laboratory under the modified protocol of Schwertmann and Cornell (2008). The Fe (oxyhydr)oxide named FoF was prepared according to the protocol for ferrihydrite, by dissolving 40 g Fe(NO_3_)_3_ 9H_2_O in 500 mL distilled water and adding 330 mL of 1 M KOH to adjust the pH to 7–8. The mixture was centrifuged at 5,000 rpm for 10 min and the supernatant was subsequently removed. The solid fraction was then washed five times with Milli-Q water. The Fe (oxyhydr)oxide named FoL was prepared according to the protocol for lepidocrocite with 11.93 g of unoxidized FeCl_2_⋅4H_2_O salts dissolved into 300 mL distilled water by stirring. The solution was adjusted to pH 6.7–6.9 with NaOH using a pH-stat under aeration (100 mL/min air). Washing was performed as described for FoF. Both synthesized minerals were freeze-dried. Goethite from Sigma-Aldrich (CAS No. 20344-49-4) and hematite from VWR Chemicals (CAS No. 1309-37-1) were also used. Mineralogical morphologies of all Fe (oxyhydr)oxides were characterized using a scanning electron microscope (SEM) and Brunauer, Emmett and Teller (BET) surface area measurement (determined by multipoint BET N_2_ adsorption) (Brunauer et al., 1938). Specific surface areas were determined from N_2_ adsorption isotherms in the best linear range (with a minimum of 15 points) between the relative pressure P/Po 0.03 and 0.33 (Cavelan et al., 2019).

Specific surface areas of Fe (oxyhydr)oxides varied from 11.7 to 337 m^2^ g^–1^ (Table 1), and compared well to some other synthetic (oxyhydr)oxides (Larsen and Postma, 2001; Bonneville et al., 2004; Pedersen et al., 2005; Das et al., 2013). SEM was performed on a TM 3000 coupled to a SwiftED3000 X-Stream module (Hitachi), and operated at 15 kV accelerating voltage (Thouin et al., 2016). The corresponding observed morphologies (Supplementary Figure S2) are given in Table 1.

**TABLE 1:** Characteristics of Fe(III) oxides submitted to Fe-reducing bacteria.

Iron oxide	Assumed morphology^a^	Surface area^b^ (m^2^g^–1^)
goethite	Acicular	11.7
hematite	cylinder/rod	31.4
FoF	Blocky	232
FoL	Blocky	337

*^a^For use in estimating mean particle size from morphology by SEM-EDS [TM3000 accompanied by a SwiftED3000 X-Stream module (Hitachi)]. ^b^Determined by multipoint BET N_2_ adsorption.*

### IRB Incubation Experiments

Incubation experiments were performed in 50 mL glass bottles containing 50 mL medium, equipped with chlorobutyl rubber stoppers, using 10% (v/v) of inocula from D1, D2, and D3 [see section “Soil and Sediment Sampling and Enrichment of Iron-Reducing Bacteria (IRB)”] enriched from the site samples of Decize and the four Fe (oxyhydr)oxides presenting contrasting specific surfaces (FoF, FoL, goethite, and hematite), under anaerobic conditions. The compositions of the different solutions used to prepare the culture medium were the same as for the enrichment cultures and are also listed in Supplementary Figure S1. The total Fe(III) concentration added as Fe (oxyhydr)oxides was adjusted to be close to 20 mM, as Fe(III)-NTA or solid iron oxides, based on the theoretical formula of each (Supplementary Table S1). The inoculation of Fe-reducing cultures was performed in an anaerobic glove box. The gas phase of the bottles was N_2_ and the bottles were flushed with N_2_ before and after sampling. The flasks were incubated at 20°C, under agitation (100 rpm). Not inoculated controls were prepared in the same conditions. Samples (1.5–2 mL) were collected as described in section “Soil and Sediment Sampling and Enrichment of Iron-Reducing Bacteria (IRB)” and analyzed for total dissolved iron ([FeT]_D_). The Fe solubilization rates were calculated using the data of [FeT]_D_ collected during the first phase of the incubation, when this parameter increased linearly. After 27 days incubation, the remaining cultures were centrifuged at 5,000 rpm for 10 min. Supernatants were removed and solids freeze-dried for observation under SEM-XEDS.

### Fe Analyses and pH/Eh

For Fe analyses, 1.5 mL aliquot was sampled with a syringe and filtered through a 0.2 μm filter into 5 mL tubes and immediately acidified with concentrated HCl in the glove box. [FeII]_D_ (dissolved Fe(II) concentration) was determined using the ortho-phenanthroline colorimetry method (Murti et al., 1966; Mamindy-Pajany et al., 2013). [FeT]_D_ was determined using the same method but with the addition of hydroquinone to reduce dissolved total ferric iron ([FeIII]_D_) into total ferrous iron ([FeII]_D_). pH and redox potential (Eh, ref. Ag/AgCl) were measured in samples taken from the incubation flasks using standard hand-held portable meters (WTW Multi340i set) in glove box before and after the incubation.

### DNA Extraction and qPCR of 16S rRNA Genes

DNA extractions were performed on all samples at the end of the experiments. Biomass was harvested by centrifugation at 10,000 rpm for 10 min of 2 mL of culture. Microbial DNA was extracted using the Fast DNA^TM^ SPIN Kit for Soil (MP Biomedicals, United States) according to the manufacturer’s instructions. The integrity of the DNA products was checked with agarose gel electrophoresis. The DNA concentrations were determined with a Quantus^TM^ Fluorometer (Promega, United States).

Quantification of *Shewanella* and *Geobacter* were performed by quantitative PCR (qPCR) of a fragment of the gene encoding 16S rRNA (*rrs* gene) (abbreviated *Shewanella* 16S and *Geobacter* 16S), using a CFX96 Touch^TM^ Real-Time PCR Detection System (Bio-Rad, United States). Primers Sw 640-F (5′-RAC TAG AGT CTT GTA GAG G-3′) and Sw 815-R (5′-AAG DYA CCA AAY TCC GAG TA-3′) specific to *Shewanella rrs* gene (Snoeyenbos-West et al., 2000; Li et al., 2018), and primers Geo 564-F (5′-AAG CGT TGT TCG GAW TTA T-3′) and Geo 840-R (5′-GGC ACT GCA GGG GTC AAT A-3′) specific to *Geobacter rrs* gene (Kim et al., 2012) were used. qPCR reactions were performed in a total volume of 20 μL containing: 7.68 μL of sterile nuclease- and nucleic acids-free water, 10 μL of SSO Advanced Universal SYBR Green Supermix (Bio-Rad), 0.16 μL of each primer at 50 μM, and 2 μL of DNA (1–5 ng⋅μL^–1^). qPCR reaction programs were as follows: for *Shewanella*, 1 min at 95°C, followed by 40 cycles: 5 s at 95°C/30 s at 55°C/30 s at 72°C/30 s at 80°C; for *Geobacter*, 2 min at 95°C, followed by 45 cycles: 10 s at 95°C/20 s at 60°C/20 s at 72°C/30 s at 80°C. Plasmid DNA containing the target genes were constructed from *Shewanella putrefaciens* and *Geobacter metallireducens rrs* gene, PCR amplified with primers 640F/815R and 564F/840R, respectively, and cloned using the TOPO^TM^ TA Cloning^TM^ Kit for Sequencing (Invitrogen, United States) according to the instructions. A calibration curve was obtained from serial dilutions of a known quantity of linearized plasmids containing known copy numbers of *S. putrefaciens* or *G. metallireducens rrs* genes. All samples, controls and standards were analyzed in duplicates. Results were reported as gene copies per gram or microliter of culture. Generation of a specific PCR product was confirmed by DNA melting curve analysis and agarose gel electrophoresis.

Quantification of the bacterial *rrs* gene coding 16S rRNA (abbreviated bacterial 16S), was performed with primers 341-F (5′-CCT ACG GGA GGC AGC AG-3′) and 515-R (5′-TGC CAG CAG CCG CGG TAA T-3′), as described for *Shewanella* and *Geobacter* except 0.2 μL T4GP32 at 500 ng⋅μL^–1^ was added into the reaction mixture. qPCR reaction program was as follows: 3 min at 95°C, followed by 35 cycles: 30 s at 95°C/30 s at 60°C/30 s at 72°C/30 s at 80°C. Plasmid DNA containing the bacterial *rrs* gene of *Pseudomonas putida* KT 2440 was 10-fold serially diluted to obtain a calibration curve of known copy numbers of *P. putida* KT 2440 *rrs* gene.

### CE-SSCP Fingerprints

Capillary Electrophoresis-Single Strand Conformational Polymorphism (CE-SSCP) (Delbès et al., 2000) profiles were performed in order to characterize the bacterial community structure in cultures. About 200 bp of the V3 region of the bacterial *rrs* gene was amplified from DNA extracts with the forward primer w49 (5′-ACGGTCCAGACTCCTACGGG-3′; *Escherichia coli* position, 331) and the reverse primer w34 (5′-TTACCGCGGCTGCTGGCAC-3′; *E. coli* position, 533), 5′end labeled with the fluorescent dye FAM, using 25 cycles, hybridization at 61°C, and 30 s elongation at 72°C. 1 μL of diluted (20 or 50 fold in nuclease-free water) PCR product was added to a mixture of 18.6 μL of deionized formamide and 0.4 μL of Genescan-600 LIZ internal standard (Applied Biosystems). To obtain single-strand DNA, samples were heat-denaturized for 5 min at 95°C, and immediately cooled on ice. CE-SSCP analyses were performed on an ABI Prism 310 genetic analyzer using a 47-cm length capillary, a non-denaturing 5.6% CAP polymer (Applied Biosystems) and the following electrophoresis conditions: run temperature 32°C, sample injection for 5 s at 15 kV, data collection for 35 min at 12 kV. CE-SSCP data analyzes and lining CE-SSCP profiles up to the internal standard and to a common baseline were performed using BioNumerics V7.5 (Applied Maths).

### Determination of Iron Oxides Solubilisation Parameters

The total dissolved Fe (Fe solubilization) was calculated from the [FeT]_D_ curves during all the incubation period for the batch experiments. The initial Fe reduction rate (mg L^–1^⋅h^–1^) was calculated for the period of rapid increase of [FeT]_D_ during the first stage (3–8 days) of the batch experiments. The total dissolved Fe and initial Fe (oxyhydr)oxide dissolution rates are indicated as “Fe solubilization” and “solubilization rate” in the following statistics.

### Statistics

DNA quantification and qPCR data were analyzed using a Kruskal–Wallis test with XLSTAT software (version 2019 21.1.3) to determine the significant differences between each culture or between iron oxides. Variations in bacterial community structure were further analyzed by Non-Metric multiDimensional Scaling (nMDS) and ANOSIM analysis applied to a Bray-Curtis dissimilarity matrix of CE-SSCP data (generated in BioNumerics V7.5), using R-Studio software (Vegan Package) (R Studio Team, 2015).

Principal component analysis (PCA) was used to summarize the relationships between chemical (Fe-reducing speed for the first few days and Fe reduction proportion) and microbial (molecular biomass, i.e., DNA concentration, and *Geobacter* and *Shewanella* gene abundances) data with XLSTAT software (version 2019 21.1.3).

## Results

### Dissolution of Fe (Oxyhydr)oxides

**FIGURE 1:**
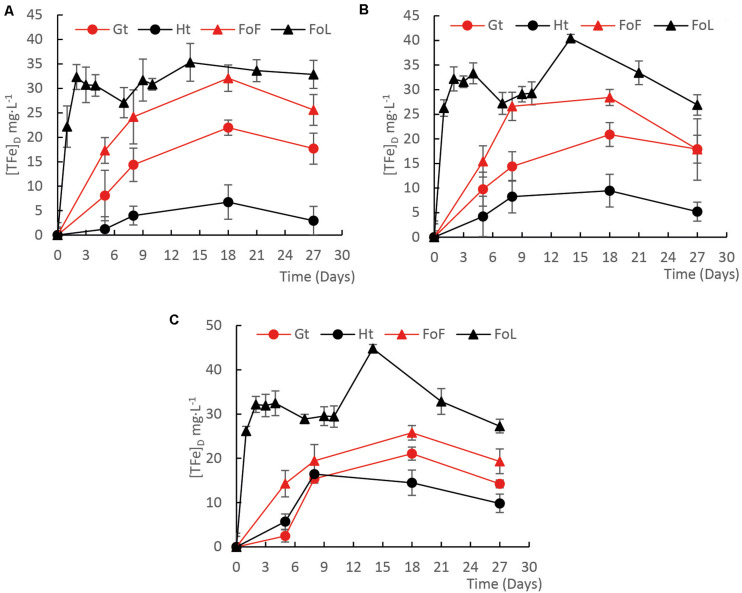
Evolution of the concentration of total Fe during incubation experiments with four Fe(III) (oxyhydr)oxides in presence of D1 **(A)**, D2 **(B)**, and D3 **(C)** iron-reducing cultures with a mixture of C sources, *Fe(III)-NTA* is given in Supplementary Figure S3. Error bars represent the standard deviation of triplicate measurements.

Fe (oxyhydr)oxide solubilization in the incubations was mainly influenced by the type of Fe (oxyhydr)oxide. For all cultures, D1 (Figure 1A), D2 (Figure 1B), and D3 (Figure 1C), the highest iron solubilization rates were observed in presence of FoL. The iron solubilization rates during the first week of the experiment, regardless of mineral structure, roughly matched the order of specific surface area except for goethite/hematite with D1 and D2 (Supplementary Table S2). In abiotic control flasks, iron dissolution remained lower than 0.4% (Supplementary Figure S3).

Initial pH of the medium was close to 7.5. This parameter did not significantly change during the incubation in presence of FoL and goethite. The final pH increased slightly to 7.6 in presence of FoF and decreased to 7.4 in presence of hematite. The initial Eh was −30 mV (ref. Ag/AgCl). This parameter decreased to −230 ± 10 mV after incubation in presence of bacteria. In abiotic control flasks, the Eh value decreased down to −130 mV.

**FIGURE 2:**
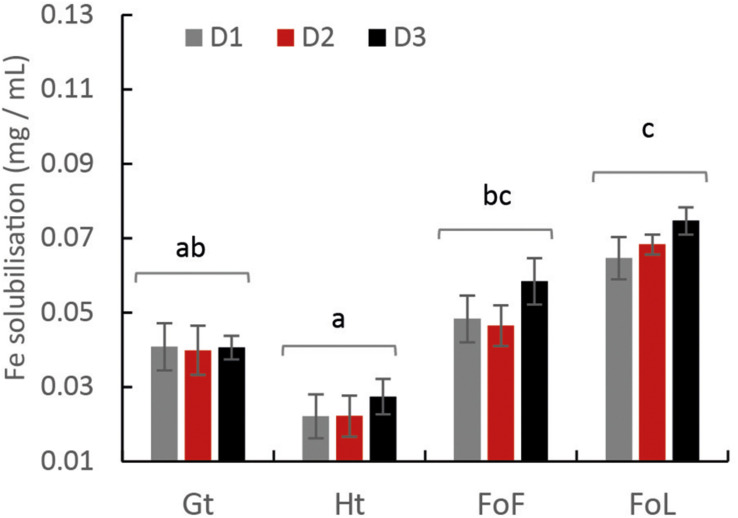
Final total amount of solubilized Fe from Fe (oxyhydr)oxides: goethite, hematite, FoF and FoL in presence of D1, D2, and D3 inocula with a mixture of C sources. The letters “a” and “b” represent the significance of differences (Kruskal–Wallis test at *p* < 0.05) between cultures. Values in the same Fe (oxyhydr)oxides group are not significantly different from one another. Error bars represent the standard deviation of triplicate measurements.

According to the [FeT]_D_ during the incubation period, total dissolved Fe from Fe (oxyhydr)oxides was calculated (Figure 2). Considering that total Fe provided as Fe minerals was close to 20 mM, the percentage of Fe solubilization was in the range from 1 to 15%. The highest quantities of dissolved Fe were obtained for FoL, with 0.074 mg iron per mL with D3 inoculum. The extent of FoL solubilization was more than three times higher than that of hematite with the same inoculum. For goethite, low solubilization, around 0.022 mg iron per mL, were obtained, with no significant differences between the three inocula. Moreover, when Fe (oxyhydr)oxides are grouped without distinguishing the origin of the inocula, the solubilization of FoL was significantly different to that of goethite and hematite (Figure 2), and that of FoF was significantly different to that of hematite. For the same data set, the significant differences shown between the different inocula (Supplementary Figure S4) shows that for both D1 and D2, FoL solubilization was significantly different to that of hematite. However, there was no significant difference in iron solubilization between iron oxides with D3. This suggests that D3 could be less influenced by Fe mineralogy than the other two inocula.

### Biological Parameters

#### Soil Samples

Globally, *Geobacter* was clearly present in higher proportions than *Shewanella* in the bacterial communities present in the environmental samples used to enrich D1, D2, and D3.

#### Effect of Fe (Oxyhydr)oxides on Bacterial Community Structure

The bacterial community structure profiles of the initial soil and sediment samples, the Fe-NTA enrichments and the cultures in presence of the four different (oxyhydr)oxides were compared using an nMDS ordination approach (Figure 4). Full CE-SSCP profiles are available in Supplementary Figure S5. These profiles show a high diversity with many peaks.

**FIGURE 3:**
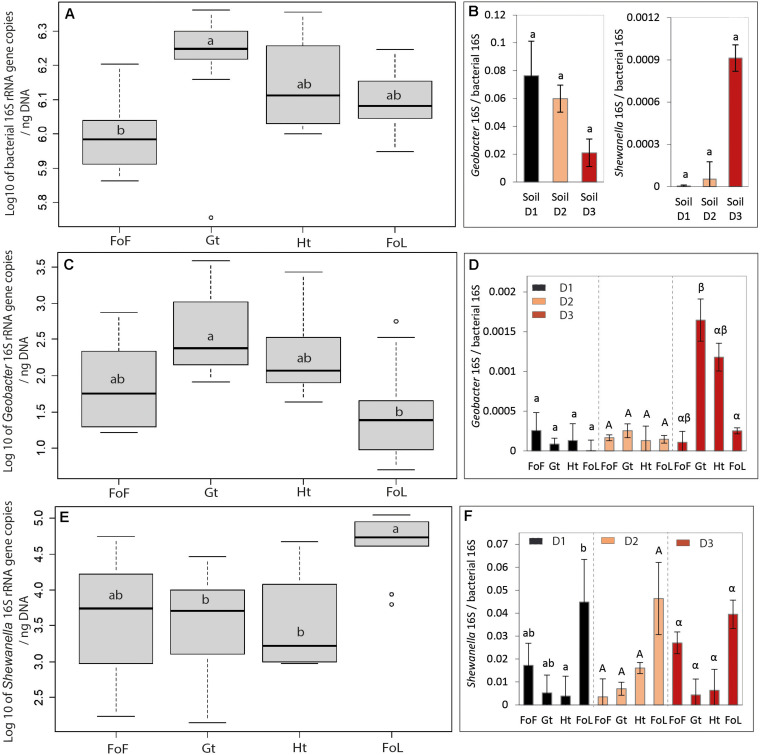
Parameters linked to bacterial abundance: **(A)** Log10 of bacterial 16S rRNA (*rrs* gene) copies, **(B)** Ratio of *Shewanella* and *Geobacter* over bacterial 16S rRNA (*rrs* gene) copies for all three site samples D1, D2, and D3, **(C)** Log 10 of *Geobacter* 16S gene copies, **(D)** Ratio *Geobacter* 16S over bacterial 16S rRNA (*rrs* gene) copies, **(E)** Log 10 of *Shewanella* 16S gene copies, and **(F)** Ratio *Shewanella* 16S over bacterial 16S rRNA (*rrs* gene) copies. Details of bacterial, *Shewanella* and *Geobacter* 16S rRNA (*rrs* gene) copies for all three site samples D1, D2, and D3 are given in Supplementary Figure S6. The letters “a” and “b” differed significantly (Kruskal–Wallis test at *p* < 0.05) between group of iron oxide for graph “(a), (b), (c), and (e)”; the small letter, capital letter and Greek letter were used for differing significantly by group of inocula D1, D2, and D3 for graphs “(d) and (f).” Data represent average values of three experimental replicates and their standard deviation (σ) for graph “(b), (d), and (f),” 3 inocula × 3 replicates for graphs “(a), (c), and (e).” Ht: hematite; Fh: FoF; Lp: FoL; Gt: goethite. Error bars represent the standard deviation of triplicate measurements.

**FIGURE 4:**
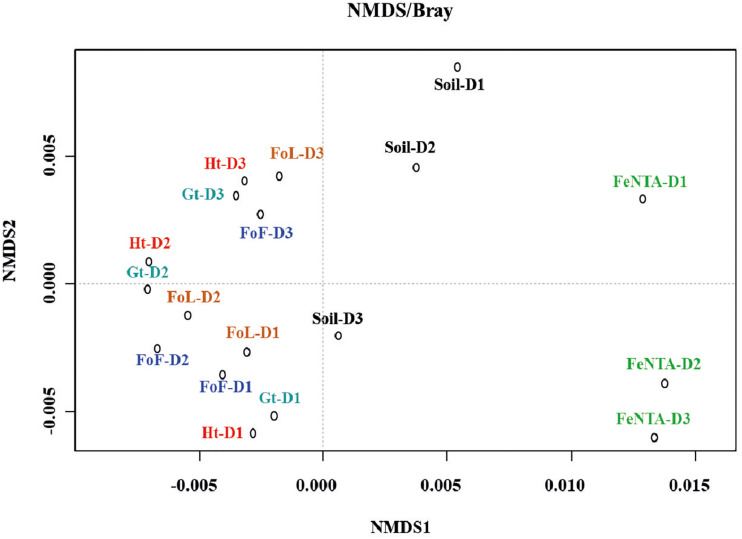
nMDS ordination of D1, D2, and D3 community fingerprints applied to a Bray-Curtis dissimilarity matrix. Plot stress = 0.15. Ht: hematite; Fh: FoF; Lp: FoL; Gt: goethite.

The structures of the initial communities were modified after inoculation on solid Fe (oxyhydr)oxides (Figure 4): enrichments on Fe(III)-NTA are grouped on the right side of the representation, whereas communities obtained by cultures with iron oxides are gathered on the left side. The three initial communities from environmental samples are located between these two poles. No clear separation is observed between the communities obtained with the four pure iron oxides. Although, according to ANOSIM analysis, no significant difference in community composition was found between initial inocula (significance > 0.05), a significant dissimilarity was found between community origins (i.e., initial soils and form of Fe(III) provided to the consortia) (*R* = 0.345 and significance = 0.0249).

#### Bacterial Abundance and Abundances of *Shewanella* and *Geobacter*

The *rrs* gene abundances and *Geobacter* 16S, *Shewanella* 16S/bacterial 16S ratios did not highlight any significant differences between the three initial soils D1, D2, and D3 (Figure 3B). As shown in Figure 3A, globally, the *rrs* gene abundance in goethite samples was significantly different to FOF samples. Conversely, the abundance of the two quantified IRB, i.e., *Geobacter* and *Shewanella*, differ between the types of Fe oxides provided as Fe(III) source (Figures 3C,E). Globally, compared with *Geobacter*, *Shewanella* was present in a larger proportion in all bacterial communities with the four iron oxides. Moreover, the number of gene copies of *Shewanella* for FoL is significantly different in D1 from that in presence of other iron oxides but there are no significant differences between the cultures for FoF, goethite and hematite samples (Figure 3F). When the type of inocula is not taken into account, the proportion of *Shewanella* 16S genes for FoL samples is significantly different for goethite and hematite samples (Figure 3E).

Considering specifically each quantified genus, the cultures in presence of goethite inoculated with D3 bacteria contained a significantly different proportion of *Geobacter* 16S genes than the same culture (D3) with FoL (Figure 3F). In the other conditions (D1, D2 with all oxides), no significant differences between the proportion of *Geobacter* 16S genes in the global bacterial population was observed.

## Discussion

### Influence of the Type of Iron Oxide on Bacterial Iron Solubilisation

FoL and FoF were synthetized in the laboratory and were more amorphous, thus more reactive, than goethite and hematite (Usman et al., 2012). According to the literature, abiotic rates of reductive iron dissolution are correlated with the solubility of Fe (oxyhydr)oxides (Larsen and Postma, 2001). The rates of this abiotic reductive bulk dissolution decrease according to ferrihydrite > lepidocrocite > goethite > hematite, emphasizing the importance of the crystal structure on the dissolution rate (Larsen and Postma, 2001). However, Roden (2006) found that in presence of IRB, oxides’ mineralogical and thermodynamical properties exert a minor influence on reduction rates compared with the abundance of available oxide surface sites controlled by oxide surface areas and the accumulation of surface-bound biogenic Fe(II). This last process, of the precipitation of new Fe(II) minerals (Urrutia et al., 1998; Zachara et al., 2002), could explain the late decrease of soluble Fe (Figure 1). Present results are partially in accordance with this last hypothesis, as Fe solubilization effectiveness increased with the specific surface, in particular for the two freshly synthesized oxides. Here, the fresh mineral prepared with the protocol of lepidocrocite (FoL) synthesis presented a higher specific surface than the mineral produced using the protocol for ferrihydrite (FoF). Synthetic lepidocrocites can present a very large range of specific surface areas, depending on their level of crystallinity (Schwertmann, 1973). In the present case, the specific area of synthetic FoL (337 m^2^⋅g^–1^) is much higher than for other Fe (oxyhydr)oxides, possibly due to a rapid oxidation of Fe(II) during synthesis that produced poor crystallization and formation of lepidocrocite impurities (Schwertmann and Taylor, 1979). Schwertmann (1973) showed that the specific surface of lepidocrocite increases with its solubility in presence of oxalate, and this specific surface area is anti-correlated with the crystallinity. In the present experiment, at the end of the incubation, we detected 8 mg⋅L^–1^ total Fe in the FoL abiotic control, but almost no Fe in FoF abiotic control. This supports the idea that FoL was more soluble than FoF. The high specific surface area and probable poor crystallinity of FoL could have favored solubilization by IRB.

Fe solubilization rates were limited after a few days, probably due to evolution of the composition of the liquid medium, or the accumulation of surface-bound biogenic Fe(II) (Roden, 2006), as Fe(III) was not the limiting factor. Parameters such as humic substances, quinones, and organic carbon can strongly influence microbial Fe(III) reduction rates for *Shewanella* (Adhikari et al., 2017) or *Geobacter* (Wolf et al., 2009) in natural environments. Generally, in these studies, the highest reducing rates were observed during the first few days of microbial Fe(III) reduction. In our study, the influence of the type of Fe (oxyhydr)oxides on initial iron oxide-reducing rates were consistent with those of previous studies performed with pure strains.

### Bacterial Communities

The structures of the initial bacterial communities present in the environmental samples were modified by the enrichment in Fe(III)-NTA medium, and again showed an evolution when the enrichments were grown in presence of solid Fe (oxyhydr)oxides (Figure 3). This last result could be due to the difference of bio-availability of Fe with minerals compared to Fe(III)-NTA. Cai et al. (2019) also showed an influence of bio-available Fe(III) on microbial community structure. Here, the accessibility of Fe(III) in the Fe(III)-NTA incubations favored the iron reducing community that may have rapidly consumed available organic substrates and probably lowered the development of other bacteria. With minerals, however, Fe(III) is less accessible and competition for Fe(III) may induce changes in community structure. For example, Zhuang et al. (2011) indicated that *Rhodoferax* and *Geobacter* species were acetate-oxidizing Fe(III)-reducers that compete in anoxic subsurface environments and this competition could influence the *in situ* bioremediation of uranium-contaminated groundwater by changing diversity structure. On the other hand, no clear effect of the type of Fe mineral on the global community profile was observed. This may be linked to the culture medium composition. Indeed, the addition of diverse C sources could enable fermentative bacteria to develop without using Fe(III) for their growth. In contrast, Lentini et al. (2012) observed an effect of the type of Fe (oxyhydr)oxide, i.e., FoF, goethite or hematite, on the structure of bacterial communities in enrichment cultures using T-RFLP fingerprints. However, these authors did not use an initial Fe(III)-NTA enrichment step and did not use a sulfate-reduction inhibitor, as in our study.

### *Geobacter* and *Shewanella* 16S Genes Abundances

The relative abundance of *Geobacter* and *Shewanella* in the initial soils and sediment used as sources of IRB in our experiment could be linked to the redox conditions of the environment. Indeed, *Geobacter* and *Shewanella* were present in higher proportions in the river sediment D3, than in the flooded soil D2, itself richer than the non-saturated soil D1. This suggests that the reducing conditions in the aquatic sediment was more favorable for anaerobic bacteria, such as IRB.

*Shewanella* and *Geobacter* represented in our incubation experiments a small proportion of the total community, however we made a focus on these two genera because they are the most studied iron reducing bacteria. However, the other members of the community could contribute either directly or indirectly to the iron solubilization process. Considering the evolution of the two targeted IRB genera, namely *Geobacter* and *Shewanella*, after the enrichment with Fe(III)-NTA, the *Shewanella*/*Geobacter* abundance ratio increased significantly compared to the site samples (Supplementary Figure S6 compared with Figure 3B), suggesting that the presence of a large amount of bio-available Fe(III) in the enrichment culture medium was in favor of *Shewanella*. Another explanation for the sharp increase of *Shewanella* compared to *Geobacter* could be the composition of the culture medium in terms of organic substrates. *Geobacter* can use acetate as electron donors while performing the dissimilatory Fe reduction (Caccavo et al., 1994; Coates et al., 1996), but does not use lactate nor glucose. Conversely, some *Shewanella* species were shown to be able to use glucose and can present either respiratory or fermentative types of metabolisms (Nogi et al., 1998; Ziemke et al., 1998; Ivanova et al., 2004). Lentini et al. (2012) showed that the presence of *Geobacter* was favored by acetate whereas the growth of *Shewanella* was rather stimulated by lactate. Moreover, these authors suggested that production of acetate through incomplete degradation of lactate by *Shewanella* could benefit *Geobacter* (Hori et al., 2015). Our medium contained both acetate, formate, lactate and glucose, thus it should potentially support growth of both *Shewanella* and *Geobacter*. However, a fermentative metabolism could explain the selection of *Shewanella* over *Geobacter* in our enrichments and in all incubation conditions, because this genus can grow either using fermentation or Fe(III) reduction (Bowman, 2015). Moreover, Leyva-Díaz et al. (2017) showed that peptone was a better substrate for the growth of *Shewanella baltica* than glucose or acetate. As our medium contained 1.5 g L^–1^ peptone, this substrate could also favor the growth of bacteria belonging to the *Shewanella* genus.

When these communities were grown in presence of solid iron oxides, the abundance of *Shewanella* (average of D1, D2, and D3) was 112, 30, 52, and 3058 times higher than the abundance of *Geobacter*, for FoF, goethite, hematite and FoL, respectively. Moreover, we found that the cultures in presence of goethite inoculated with D3 bacteria contained higher proportions of *Geobacter* than the same culture (D3) with FoL. These results might suggest that *Geobacter* could be more favored, in the competition with other IRB such as *Shewanella*, for growth in presence of goethite and hematite than for growth in presence of the more easily dissolved oxides, i.e., FoF and FoL. The type of Fe mineral can exert a selection pressure on the communities of IRB, as previously shown by Lentini et al. (2012). This result could be linked to a higher affinity of *Geobacter* for Fe(III), that would favor this organism at low Fe(III) availability levels. Reported Ks values for Fe(III) are 1.0 mM with *Geobacter sulfurreducens* (Esteve-Núñez et al., 2005) compared to 29 mM with *S. putrefaciens* (Liu et al., 2001).

Yet, the solubility of ferrihydrite and lepidocrocite are higher than that of goethite and hematite (Cornell and Schwertmann, 2003; Liu et al., 2007), thus the availability of Fe(III) could be higher with the first two oxides. *Shewanella* might be favored by high bio-available Fe(III), but could be less efficient for growth in presence of less soluble Fe-oxides such as goethite and hematite. The anaerobic respiration of *Shewanella* was highly dependent on electron shuttles. Nevin and Lovley (2002) suggested that *Shewanella alga* strain BrY released compounds that could solubilize Fe(III) from Fe(III) oxides, however, *G. metallireducens* did not produce electron shuttles or Fe(III) chelators to solubilize Fe(III) oxides (Nevin and Lovley, 2002). Kotloski and Gralnick (2013) determined the contribution of flavin electron shuttles in extracellular electron transfer by *Shewanella oneidensis* and Wu et al. (2020) showed that exogenous electron mediators (EMs) favored high density current production and increased the synthesis of extracellular polymeric substances which promoted biofilm formation during electron shuttling process (Wu et al., 2020). The conduction of electrons along pili or other filamentous structures is one of the mechanisms proposed for electron transfer to solid iron oxides. Leang et al. (2010) showed that OmcS, a cytochrome that is required for Fe(III) reduction by *G. sulfurreducens*, was localized along the pili (Leang et al., 2010). The electrically conductive pili play a major role in the adaptation of *Geobacter* to perform DIR in natural environments (Liu et al., 2019). These differences in the Fe(III)-reducing mechanisms between the two species might explain their difference of affinity for Fe(III) (Liu et al., 2001; Esteve-Núñez et al., 2005) and the relative increase of *Geobacter* abundance, observed here in presence of goethite and hematite, with the enrichment culture that was the most efficient for Fe-oxide solubilization, i.e., culture D3.

### Relation Between Iron Solubilisation Effectiveness and *Geobacter* and *Shewanella* Abundances

In batch experiments, the three inocula D1, D2, and D3, enriched from soil from the river bank, flooded soil and an aquatic sediment of the Decize site gave similar results in terms of Fe solubilization effectiveness of iron (oxyhydr)oxides. However, there were differences between different Fe (oxyhydr)oxides: the type of Fe (oxyhydr)oxides had more influence on Fe solubilization effectiveness than the origin of the inocula. Fe solubilization rates were slower with goethite and hematite compared to FoF and FoL. The same tendency was observed by Bonneville et al. (2004), who found that, with the pure strain *S. putrefaciens* in presence of 20 mM Fe(III), reduction of 6-line ferrihydrite was faster than that obtained with lepidocrocite, and faster than that obtained with low surface area hematite. Li et al. (2012) reported similar results for the microbial reduction of Fe(III) oxides by *Shewanella decolorationis* strain S12, the reducing speed decreasing according to the following order: lepidocrocite > goethite > hematite.

Principal component analysis integrating chemical (Supplementary Table S2) and molecular data was performed to identify the relationships and contributions between iron solubilization effectiveness and *Geobacter*/*Shewanella* abundances in batch experiment samples (Mercier et al., 2014; Zhu et al., 2014; Hong et al., 2015). In this study, a biplot (Supplementary Figure S7) summarizes PCA results. The first principal component is strongly influenced by the iron solubilization effectiveness (higher on the right than on the left side of the representation), with higher values associated with FoL, and the lowest associated with goethite and hematite (not separated), FoF being in an intermediary position. Meanwhile proportion of *Shewanella* in the bacterial community, but not to the proportion of *Geobacter*, seems to be correlated to FoL. The second principal component reflects high values of the proportion of *Geobacter* in the bacterial communities. Thus, the proportion of *Shewanella* seems to be more correlated to iron solubilization parameters than *Geobacter*. PCA allows clear discrimination with different groups of iron oxides, however not for the different types of inoculum (D1, D2, and D3).

According to Bonneville et al. (2004), the dissolution and solubility of goethite and hematite are lower than that of ferrihydrite and lepidocrocite in the presence of *Shewanella*. Cutting et al. (2009) indicated that hematite and goethite are susceptible to limited Fe(III) reduction in presence of *G. sulfurreducens*. Moreover, Poggenburg et al. (2016) mentioned that Fe(III)-organic compounds (coprecipitates from solutions of FeCl_3_ and natural organic matter) reduction by *S. putrefaciens* was influenced by the amount of available electron shuttling molecules induced by sorbed natural organic matter. Fe(III)-organic compounds’ reduction by *G. metallireducens* was more influenced by particle size, physicochemical properties and iron (oxyhydr)oxides (composition of sorbed natural organic matter and aggregation state) (Poggenburg et al., 2016). In our study, FoL samples with a higher proportion of *Shewanella* in their bacterial communities were correlated with high initial iron solubilization rates and electron shuttling molecules might have a role in this phenomenon. In contrast, *Geobacter* was not specifically associated to FoL but was found in higher proportions with goethite in one condition. This tendency is in accordance with findings of previous research performed with the less soluble iron oxides (Crosby et al., 2007), showing that *G. sulfurreducens* reduced 0.7% hematite and 4.0% goethite while *S. putrefaciens* reduced only 0.5% of hematite 3.1% goethite after 280 days of incubation. Thus, our results suggest that *Geobacter* might suffer less from the competition with *Shewanella* in low bio-available Fe(III) conditions, whereas the contribution of this genus, present in lower quantities than *Shewanella*, to the iron solubilization effectiveness is not demonstrated. Moreover, other members in the community of IRB might have potential contribution to Fe solubilization. Our culture medium contained substrates such as glucose and peptone that could support the growth of fermentative organisms. Lentini et al. (2012) and Gagen et al. (2019) indicated that fermentation likely plays a key role in reduction of crystalline iron oxides by diverse species, such as *Telmatospirillum*, both through direct reduction and by the production of H_2_, potentially used by dissimilatory iron reducers, during fermentation.

## Conclusion

Microbial enrichments containing IRB, obtained with a mixture of simple and complex electron donors, were able to grow and reduce Fe(III) in a short time. Experiments performed with fresh Fe (oxyhydr)oxides, goethite and hematite confirmed that the type of Fe mineral could influence Fe solubilization rates and the abundances of two IRB commonly involved as pure strains in Fe-reducing experiments, i.e., *Geobacter* and *Shewanella*.

The present study’s results showed that: (1) the sub-culturing of IRB enrichments from Fe(III)-NTA to pure iron oxides significantly modified the bacterial communities; (2) in our experimental conditions, bacterial diversity was not significantly different from one type of pure (oxyhydr)oxide to another; (3) the type of Fe oxide can influence the proportion of *Geobacter* and *Shewanella*. Meanwhile, the nature of Fe (oxyhydr)oxides seems to have exerted a selection on the ratio of *Geobacter* and *Shewanella*, whereas it did not impact the bacterial community fingerprints. The concentration of bio-available Fe(III) and the mixture of electron donors in the enrichment medium favored the development of *Shewanella* compared with *Geobacter* genus. However, the culture medium included a large amount of electron donors that is not representative of most natural systems. Therefore, complementary studies, performed with lower concentrations of electron donors provided in continuous feeding conditions would help to make the link with real environments. In presence of iron oxides, the highest proportions of *Shewanella* in bacterial communities were obtained with FoL and corresponded to the highest levels of iron solubilization, possibly linked to the fact that FoL was the most soluble (oxyhydr)oxide in our experiments. This result is consistent with the hypothesis that *Shewanella* development could be favored by a high bioavailability of Fe(III). In contrast, *Geobacter* was detected in higher proportions with goethite that is less easily dissolved, when D3 culture was used.

Globally, all results suggested that both initial community composition of the sample used to prepare the enrichments, as well as the type of Fe(III) oxide used as electron acceptor influenced the final proportions and abundances of *Geobacter* and *Shewanella*. A better knowledge of complementary biological parameters associated with these two organisms, such as their activity during Fe(III) solubilization and reduction in complex communities and distribution between planktonic and Fe(III)-mineral-attached cells, could help to elucidate their role in natural environments. As biofilms in soils and sediments contain a large part of the bacterial biomass, future research could be focused on the distribution and activity of *Geobacter* and *Shewanella* attached on iron oxides surfaces.

## Data Availability Statement

The original contributions presented in the study are included in the article/Supplementary Material, further inquiries can be directed to the corresponding author.

## Author Contributions

FB-B, JH, and MM-H conceived and designed the experiments and arranged funds. FZ performed major experiments. FZ and FB-B were responsible for manuscript preparation. MM-H, FB-B, JH, and FZ arranged sampling from Decize, France. PG and FZ performed SEM. CJ, JH, and FZ performed the qPCR of *Shewanella*/*Geobacter* and bacterial 16S rRNA (*rrs* gene) quantification. All authors contributed to the article and approved the submitted version.

## Conflict of Interest

The authors declare that the research was conducted in the absence of any commercial or financial relationships that could be construed as a potential conflict of interest.

## Supplementary Material

Supplementary Figure 1 Design and initial compositions of the batch experiments.

Supplementary Figure 2 SEM observations of iron oxides: Morphology of initial FoF **(A)**, FoL **(B)**, hematite **(C)**, and goethite **(D)**.

Supplementary Figure 3 Concentration of Fe(II) and total Fe (FeT) of D1, D2, and D3 incubated on Fe(III)-NTA medium **(A)**, and evolution of the concentration of total Fe during incubation experiments with four abiotic Fe(III) (oxyhydr)oxides **(B)**. Error bars represent the standard deviation of triplicate measurements.

Supplementary Figure 4 Dissolution of Fe(III) oxides: goethite, hematite, FoF and FoL in presence of D1, D2, and D3 inocula. The small letters, capital letters and Greek letters were used for differing statistical significance (Kruskal–Wallis test at *p* < 0.05) per group of inocula D1, D2, and D3 for the different iron oxides. Error bars represent the standard deviation of triplicate measurements.

Supplementary Figure 5 CE-SSCP diversity profiles of the site samples (Soil), the Fe(III)-NTA enrichments (Fe-NTA) and cultures in presence of the four iron oxides: FoF, FoL, goethite (Gt) and hematite (Ht). D1: soil from river bank; D2: sediment interface, flooded soil; D3: sediment under water.

Supplementary Figure 6 Parameters linked to bacterial abundance: **(A)** Log10 of bacterial 16S rRNA (*rrs* gene) copies, **(B)** Log 10 of *Geobacter* 16S gene copies, **(C)** Log 10 of *Shewanella* 16S gene copies, for the three site samples D1, D2, and D3; Ratios of *Shewanella* 16S **(D)** and *Geobacter* 16S **(E)** over bacterial 16S rRNA genes copies in Fe(III)-NTA enrichments. Error bars represent the standard deviation of triplicate measurements.

Supplementary Figure 7 Principal component analysis (F1 × F2) biplot map generated from iron solubilization (percentage and rate of iron solubilization) and ratios of *Shewanella* (*Shewanella* 16S/bacterial 16S) and *Geobacter* (*Geobacter* 16S/bacterial 16S) in the bacterial community, obtained for the FoF, goethite hematite and FoL incubations with D1, D2, and D3.

## References

[B1] Adhikari D. Zhao Q. Das K. Mejia J. Huang R. Wang X. (2017). Dynamics of ferrihydrite-bound organic carbon during microbial Fe reduction. *Geochim. Cosmochim. Acta* 212 221–233. 10.1016/j.gca.2017.06.017

[B2] Aino K. Hirota K. Okamoto T. Tu Z. Matsuyama H. Yumoto I. (2018). Microbial communities associated with indigo fermentation that thrive in anaerobic alkaline environments. *Front. Microbiol.* 9:2196. 10.3389/fmicb.2018.02196 30279681 PMC6153312

[B3] Bonneville S. Van Cappellen P. Behrends T. (2004). Microbial reduction of iron (III) oxyhydroxides: effects of mineral solubility and availability. *Chem. Geol.* 212 255–268. 10.1016/j.chemgeo.2004.08.015

[B4] Bowman J. P. (2015). Shewanella. Bergey’s Manual of Systematics of Archaea and Bacteria. New Jersey: John Wiley & Sons, Inc., 1–22.

[B5] Brunauer S. Emmett P. H. Teller E. (1938). Adsorption of gases in multimolecular layers. *J. Am. Chem. Soc.* 60 309–319. 10.1021/ja01269a023

[B6] Caccavo F. Lonergan D. J. Lovley D. R. Davis M. Stolz J. F. McInerney M. J. (1994). *Geobacter sulfurreducens* sp. nov., a hydrogen-and acetate-oxidizing dissimilatory metal-reducing microorganism. *Appl. Environ. Microbiol.* 60 3752–3759. 10.1128/aem.60.10.3752-3759.1994 7527204 PMC201883

[B7] Cai Y. Hu K. Zheng Z. Zhang Y. Guo S. Zhao X. (2019). Effects of adding EDTA and Fe2+ on the performance of reactor and microbial community structure in two simulated phases of anaerobic digestion. *Bioresour. Technol.* 275 183–191. 10.1016/j.biortech.2018.12.050 30590204

[B8] Cavelan A. Boussafir M. Le Milbeau C. Rozenbaum O. Laggoun-Défarge F. (2019). Effect of organic matter composition on source rock porosity during confined anhydrous thermal maturation: example of Kimmeridge-clay mudstones. *Int. J. Coal Geol.* 212:103236 10.1016/j.coal.2019.103236

[B9] Coates J. D. Phillips E. J. Lonergan D. J. Jenter H. Lovley D. R. (1996). Isolation of *Geobacter* species from diverse sedimentary environments. *Appl. Environ. Microbiol.* 62 1531–1536. 10.1128/aem.62.5.1531-1536.1996 8633852 PMC167928

[B10] Colombo C. Palumbo G. He J.-Z. Pinton R. Cesco S. (2014). Review on iron availability in soil: interaction of Fe minerals, plants, and microbes. *J. Soils Sediments* 14 538–548. 10.1007/s11368-013-0814-z

[B11] Cooper D. C. Picardal F. F. Coby A. J. (2006). Interactions between microbial iron reduction and metal geochemistry: effect of redox cycling on transition metal speciation in iron bearing sediments. *Environ. Sci. Technol.* 40 1884–1891. 10.1021/es051778t 16570612

[B12] Cornell R. M. Schwertmann U. (2003). *The Iron Oxides: Structure, Properties, Reactions, Occurrences and Uses.* Hoboken, NJ: John Wiley & Sons.

[B13] Crosby H. A. Johnson C. M. Roden E. E. Beard B. L. (2005). Coupled Fe (II)-Fe (III) electron and atom exchange as a mechanism for Fe isotope fractionation during dissimilatory iron oxide reduction. *Environ. Sci. Technol.* 39 6698–6704. 10.1021/es0505346 16190229

[B14] Crosby H. A. Roden E. E. Johnson C. M. Beard B. L. (2007). The mechanisms of iron isotope fractionation produced during dissimilatory Fe (III) reduction by *Shewanella putrefaciens* and *Geobacter sulfurreducens*. *Geobiology* 5 169–189. 10.1111/j.1472-4669.2007.00103.x

[B15] Cutting R. Coker V. Fellowes J. Lloyd J. Vaughan D. (2009). Mineralogical and morphological constraints on the reduction of Fe (III) minerals by *Geobacter sulfurreducens*. *Geochim. Cosmochim. Acta* 73 4004–4022. 10.1016/j.gca.2009.04.009

[B16] Das S. Hendry M. J. Essilfie-Dughan J. (2013). Adsorption of selenate onto ferrihydrite, goethite, and lepidocrocite under neutral pH conditions. *Appl. Geochem.* 28 185–193. 10.1016/j.apgeochem.2012.10.026

[B17] Delbès C. Moletta R. Godon J. J. (2000). Monitoring of activity dynamics of an anaerobic digester bacterial community using 16S rRNA polymerase chain reaction–single−strand conformation polymorphism analysis. *Environ. Microbiol.* 2 506–515. 10.1046/j.1462-2920.2000.00132.x 11233159

[B18] Dhivert E. Grosbois C. Rodrigues S. Desmet M. (2015). Influence of fluvial environments on sediment archiving processes and temporal pollutant dynamics (Upper Loire River, France). *Sci. Total Environ.* 505 121–136. 10.1016/j.scitotenv.2014.09.082 25310887

[B19] Engel C. E. A. Schattenberg F. Dohnt K. Schröder U. Müller S. Krull R. (2019). Long-term behavior of defined mixed cultures of *Geobacter sulfurreducens* and *Shewanella oneidensis* in bioelectrochemical systems. *Front. Bioeng. Biotechnol.* 7:60. 10.3389/fbioe.2019.00060 30972336 PMC6445848

[B20] Esteve-Núñez A. Rothermich M. Sharma M. Lovley D. (2005). Growth of *Geobacter sulfurreducens* under nutrient−limiting conditions in continuous culture. *Environ. Microbiol.* 7 641–648. 10.1111/j.1462-2920.2005.00731.x 15819846

[B21] Fredrickson J. K. Zachara J. M. Kennedy D. W. Dong H. Onstott T. C. Hinman N. W. (1998). Biogenic iron mineralization accompanying the dissimilatory reduction of hydrous ferric oxide by a groundwater bacterium. *Geochim. Cosmochim. Acta* 62 3239–3257. 10.1016/s0016-7037(98)00243-9

[B22] Gagen E. J. Zaugg J. Tyson G. W. Southam G. (2019). Goethite reduction by a neutrophilic member of the alphaproteobacterial genus *Telmatospirillum*. *Front. Microbiol.* 10:2938. 10.3389/fmicb.2019.02938 31921089 PMC6933298

[B23] Gault A. G. Ibrahim A. Langley S. Renaud R. Takahashi Y. Boothman C. (2011). Microbial and geochemical features suggest iron redox cycling within bacteriogenic iron oxide-rich sediments. *Chem. Geol.* 281 41–51. 10.1016/j.chemgeo.2010.11.027

[B24] Ghorbanzadeh N. Lakzian A. Halajnia A. Choi U. K. Kim K. H. Kim J. O. (2017). Impact of bioreduction on remobilization of adsorbed cadmium on iron minerals in anoxic condition. *Water Environ. Res.* 89 519–526. 10.2175/106143017x14902968254449 28545603

[B25] Han R. Liu T. Li F. Li X. Chen D. Wu Y. (2018). Dependence of secondary mineral formation on Fe (II) production from ferrihydrite reduction by *Shewanella oneidensis* MR-1. *ACS Earth Space Chem.* 2 399–409. 10.1021/acsearthspacechem.7b00132

[B26] Hansel C. M. Benner S. G. Nico P. Fendorf S. (2004). Structural constraints of ferric (hydr) oxides on dissimilatory iron reduction and the fate of Fe (II). *Geochim. Cosmochim. Acta* 68 3217–3229. 10.1016/j.gca.2003.10.041

[B27] Hong C. Si Y. Xing Y. Li Y. (2015). Illumina MiSeq sequencing investigation on the contrasting soil bacterial community structures in different iron mining areas. *Environ. Sci. Pollut. Res.* 22 10788–10799. 10.1007/s11356-015-4186-3 25761991

[B28] Hori T. Aoyagi T. Itoh H. Narihiro T. Oikawa A. Suzuki K. (2015). Isolation of microorganisms involved in reduction of crystalline iron (III) oxides in natural environments. *Front. Microbiol.* 6:386. 10.3389/fmicb.2015.00386 25999927 PMC4419728

[B29] Huguet L. (2009). *Caractérisation Biogéochimique et Potentiel de Méthylation du Mercure de Biofilms en Milieu Tropical (Retenue de Petit Saut et estuaire du Sinnamary, Guyane Française).* Doctoral dissertation, Nancy 1, Nancy.

[B30] Ivanova E. P. Gorshkova N. M. Bowman J. P. Lysenko A. M. Zhukova N. V. Sergeev A. F. (2004). *Shewanella pacifica* sp. nov., a polyunsaturated fatty acid-producing bacterium isolated from sea water. *Int. J. Syst. Evol. Microbiol.* 54(Pt 4) 1083–1087. 10.1099/ijs.0.02993-0 15280273

[B31] Jiang Z. Shi M. Shi L. (2020). Degradation of organic contaminants and steel corrosion by the dissimilatory metal-reducing microorganisms *Shewanella* and *Geobacter* spp. *Int. Biodeterior. Biodegradation* 147:104842 10.1016/j.ibiod.2019.104842

[B32] Kim S.-J. Koh D.-C. Park S.-J. Cha I.-T. Park J.-W. Na J.-H. (2012). Molecular analysis of spatial variation of iron-reducing bacteria in riverine alluvial aquifers of the Mankyeong River. *J. Microbiol.* 50 207–217. 10.1007/s12275-012-1342-z 22538648

[B33] Kotloski N. J. Gralnick J. A. (2013). Flavin electron shuttles dominate extracellular electron transfer by *Shewanella oneidensis*. *MBio* 4:e00553-12. 10.1128/mBio.00553-12 23322638 PMC3551548

[B34] Kwon M. J. O’Loughlin E. J. Boyanov M. I. Brulc J. M. Johnston E. R. Kemner K. M. (2016). Impact of organic carbon electron donors on microbial community development under iron-and sulfate-reducing conditions. *PLoS One* 11:e0146689. 10.1371/journal.pone.0146689 26800443 PMC4723079

[B35] Larsen O. Postma D. (2001). Kinetics of reductive bulk dissolution of lepidocrocite, ferrihydrite, and goethite. *Geochim. Cosmochim. Acta* 65 1367–1379. 10.1016/s0016-7037(00)00623-2

[B36] Leang C. Qian X. Mester T. Lovley D. R. (2010). Alignment of the c-type cytochrome OmcS along pili of *Geobacter sulfurreducens*. *Appl. Environ. Microbiol.* 76 4080–4084. 10.1128/aem.00023-10 20400557 PMC2893476

[B37] Lentini C. J. Wankel S. D. Hansel C. M. (2012). Enriched iron (III)-reducing bacterial communities are shaped by carbon substrate and iron oxide mineralogy. *Front. Microbiol.* 3:404. 10.3389/fmicb.2012.00404 23316187 PMC3541049

[B38] Levar C. E. Hoffman C. L. Dunshee A. J. Toner B. M. Bond D. R. (2017). Redox potential as a master variable controlling pathways of metal reduction by *Geobacter sulfurreducens*. *ISME J.* 11 741–752. 10.1038/ismej.2016.146 28045456 PMC5322298

[B39] Leyva-Díaz J. C. Poyatos J. M. Barghini P. Gorrasi S. Fenice M. (2017). Kinetic modeling of *Shewanella baltica* KB30 growth on different substrates through respirometry. *Microb. Cell Fact.* 16:189. 10.1186/s12934-017-0805-7 PMC5670636 29100519

[B40] Li B.-B. Cheng Y.-Y. Fan Y.-Y. Liu D.-F. Fang C.-Y. Wu C. (2018). Estimates of abundance and diversity of *Shewanella* genus in natural and engineered aqueous environments with newly designed primers. *Sci. Total Environ.* 637-638 926–933. 10.1016/j.scitotenv.2018.05.051 29763873

[B41] Li X. Liu T. Li F. Zhang W. Zhou S. Li Y. (2012). Reduction of structural Fe (III) in oxyhydroxides by *Shewanella* decolorationis S12 and characterization of the surface properties of iron minerals. *J. Soils Sediments* 12 217–227. 10.1007/s11368-011-0433-5

[B42] Liu C. Kota S. Zachara J. M. Fredrickson J. K. Brinkman C. K. (2001). Kinetic analysis of the bacterial reduction of goethite. *Environ. Sci. Technol.* 35 2482–2490. 10.1021/es001956c 11432552

[B43] Liu H. Li P. Zhu M. Wei Y. Sun Y. (2007). Fe (II)-induced transformation from ferrihydrite to lepidocrocite and goethite. *J. Solid State Chem.* 180 2121–2128. 10.1016/j.jssc.2007.03.022

[B44] Liu X. Ye Y. Xiao K. Rensing C. Zhou S. (2019). Molecular evidence for the adaptive evolution of *Geobacter sulfurreducens* to perform dissimilatory iron reduction in natural environments. *Mol. Microbiol.* 113 783–793. 10.1111/mmi.14443 31872462

[B45] Lovley D. (2006). Dissimilatory Fe (III)-and Mn (IV)-reducing prokaryotes. *Prokaryotes* 2 635–658. 10.1007/0-387-30742-7_21

[B46] Lovley D. R. (1991). Dissimilatory Fe (III) and Mn (IV) reduction. *Microbiol. Mol. Biol. Rev.* 55 259–287. 10.1128/mmbr.55.2.259-287.1991 PMC372814 1886521

[B47] Lovley D. R. (2000). “Fe (III) and Mn (IV) reduction,” in *Environmental Microbe-Metal Interactions*, ed. Lovley D. R. (Washington, DC: American Society of Microbiology), 3–30.

[B48] Lovley D. R. Phillips E. J. Lonergan D. J. (1989). Hydrogen and formate oxidation coupled to dissimilatory reduction of iron or manganese by *Alteromonas putrefaciens*. *Appl. Environ. Microbiol.* 55 700–706. 10.1128/aem.55.3.700-706.1989 16347876 PMC184183

[B49] Lovley D. R. Stolz J. F. Nord G. L. Phillips E. J. (1987). Anaerobic production of magnetite by a dissimilatory iron-reducing microorganism. *Nature* 330 252–254. 10.1038/330252a0

[B50] Mamindy-Pajany Y. Bataillard P. Séby F. Crouzet C. Moulin A. Guezennec A.-G. (2013). Arsenic in marina sediments from the Mediterranean coast: speciation in the solid phase and occurrence of thioarsenates. *Soil Sediment Contam.* 22 984–1002. 10.1080/15320383.2013.770441

[B51] Meile C. Scheibe T. D. (2019). Reactive transport modeling of microbial dynamics. *Elements* 15 111–116. 10.2138/gselements.15.2.111

[B52] Melton E. D. Swanner E. D. Behrens S. Schmidt C. Kappler A. (2014). The interplay of microbially mediated and abiotic reactions in the biogeochemical Fe cycle. *Nat. Rev. Microbiol.* 12 797–808. 10.1038/nrmicro3347 25329406

[B53] Mercier A. Joulian C. Michel C. Auger P. Coulon S. Amalric L. (2014). Evaluation of three activated carbons for combined adsorption and biodegradation of PCBs in aquatic sediment. *Water Res.* 59 304–315. 10.1016/j.watres.2014.04.021 24813338

[B54] Murti G. K. Volk V. Jackson M. (1966). Colorimetric determination of iron of mixed valency by orthophenanthroline. *Soil Sci. Soc. Am. J.* 30 663–664. 10.2136/sssaj1966.03615995003000050037x

[B55] Nealson K. H. (2017). Bioelectricity (electromicrobiology) and sustainability. *Microb. Biotechnol.* 10 1114–1119. 10.1111/1751-7915.12834 28805347 PMC5609272

[B56] Nevin K. P. Lovley D. R. (2002). Mechanisms for Fe (III) oxide reduction in sedimentary environments. *Geomicrobiol. J.* 19 141–159. 10.1080/01490450252864253

[B57] Nogi Y. Kato C. Horikoshi K. (1998). Taxonomic studies of deep-sea barophilic *Shewanella* strains and description of *Shewanella violacea* sp. nov. *Arch. Microbiol.* 170 331–338. 10.1007/s002030050650 9818352

[B58] Pedersen H. D. Postma D. Jakobsen R. Larsen O. (2005). Fast transformation of iron oxyhydroxides by the catalytic action of aqueous Fe (II). *Geochim. Cosmochim. Acta* 69 3967–3977. 10.1016/j.gca.2005.03.016

[B59] Poggenburg C. Mikutta R. Sander M. Schippers A. Marchanka A. Dohrmann R. (2016). Microbial reduction of ferrihydrite-organic matter coprecipitates by *Shewanella putrefaciens* and *Geobacter metallireducens* in comparison to mediated electrochemical reduction. *Chem. Geol.* 447 133–147. 10.1016/j.chemgeo.2016.09.031

[B60] Roden E. E. (2006). Geochemical and microbiological controls on dissimilatory iron reduction. *C. R. Geosci.* 338 456–467. 10.1016/j.crte.2006.04.009

[B61] Roden E. E. Sobolev D. Glazer B. Luther G. W. (2004). Potential for microscale bacterial Fe redox cycling at the aerobic-anaerobic interface. *Geomicrobiol. J.* 21 379–391. 10.1080/01490450490485872

[B62] Roden E. E. Wetzel R. G. (2002). Kinetics of microbial Fe (III) oxide reduction in freshwater wetland sediments. *Limnol. Oceanogr.* 47 198–211. 10.4319/lo.2002.47.1.0198

[B63] Schilling K. Borch T. Rhoades C. C. Pallud C. E. (2019). Temperature sensitivity of microbial Fe (III) reduction kinetics in subalpine wetland soils. *Biogeochemistry* 142 19–35. 10.1007/s10533-018-0520-4

[B64] Schwertmann U. (1973). Use of oxalate for Fe extraction from soils. *Can. J. Soil Sci.* 53 244–246. 10.4141/cjss73-037

[B65] Schwertmann U. Cornell R. M. (2008). *Iron Oxides in the Laboratory: Preparation and Characterization.* Hoboken, NJ: John Wiley & Sons.

[B66] Schwertmann U. Taylor R. M. (1979). Natural and synthetic poorly crystallized lepidocrocite. *Clay Miner.* 14 285–293. 10.1180/claymin.1979.014.4.05

[B67] Shelobolina E. S. Nevin K. P. Blakeney-Hayward J. D. Johnsen C. V. Plaia T. W. Krader P. (2007). *Geobacter pickeringii* sp. nov., *Geobacter argillaceus* sp. nov. and *Pelosinus fermentans* gen. nov., sp. nov., isolated from subsurface kaolin lenses. *Int. J. Syst. Evol. Microbiol.* 57(Pt 1) 126–135. 10.1099/ijs.0.64221-0 17220454

[B68] Shi L. Dong H. Reguera G. Beyenal H. Lu A. Liu J. (2016). Extracellular electron transfer mechanisms between microorganisms and minerals. *Nat. Rev. Microbiol.* 14 651–662. 10.1038/nrmicro.2016.93 27573579

[B69] Shi M. Jiang Y. Shi L. (2019). Electromicrobiology and biotechnological applications of the exoelectrogens *Geobacter* and *Shewanella* spp. *Sci. China Technol. Sci.* 62 1670–1678. 10.1007/s11431-019-9509-8

[B70] Snoeyenbos-West O. Nevin K. Anderson R. Lovley D. (2000). Enrichment of *Geobacter* species in response to stimulation of Fe (III) reduction in sandy aquifer sediments. *Microb. Ecol.* 39 153–167. 10.1007/s002480000018 10833228

[B71] Stern N. Mejia J. He S. Yang Y. Ginder-Vogel M. Roden E. E. (2018). Dual role of humic substances as electron donor and shuttle for dissimilatory iron reduction. *Environ. Sci. Technol.* 52 5691–5699. 10.1021/acs.est.7b06574 29658273 PMC6211804

[B72] Su C. Zhang M. Lin L. Yu G. Zhong H. Chong Y. (2020). Reduction of iron oxides and microbial community composition in iron-rich soils with different organic carbon as electron donors. *Int. Biodeterior. Biodegradation* 148:104881 10.1016/j.ibiod.2019.104881

[B73] RStudio Team (2015). *RStudio: Integrated Development for R.* Boston, MA: RStudio Inc., 700.

[B74] Thompson A. Chadwick O. A. Rancourt D. G. Chorover J. (2006). Iron-oxide crystallinity increases during soil redox oscillations. *Geochim. Cosmochim. Acta* 70 1710–1727. 10.1016/j.gca.2005.12.005

[B75] Thouin H. Le Forestier L. Gautret P. Hube D. Laperche V. Dupraz S. (2016). Characterization and mobility of arsenic and heavy metals in soils polluted by the destruction of arsenic-containing shells from the Great War. *Sci. Total Environ.* 550 658–669. 10.1016/j.scitotenv.2016.01.111 26849330

[B76] Urrutia M. Roden E. Fredrickson J. Zachara J. (1998). Microbial and surface chemistry controls on reduction of synthetic Fe (III) oxide minerals by the dissimilatory iron−reducing bacterium *Shewanella alga*. *Geomicrobiol. J.* 15 269–291. 10.1080/01490459809378083

[B77] Usman M. Hanna K. Abdelmoula M. Zegeye A. Faure P. Ruby C. (2012). Formation of green rust via mineralogical transformation of ferric oxides (ferrihydrite, goethite and hematite). *Appl. Clay Sci.* 64 38–43. 10.1016/j.clay.2011.10.008

[B78] Wilkins M. J. Livens F. R. Vaughan D. J. Lloyd J. R. (2006). The impact of Fe (III)-reducing bacteria on uranium mobility. *Biogeochemistry* 78 125–150. 10.1007/s10533-005-3655-z

[B79] Wolf M. Kappler A. Jiang J. Meckenstock R. U. (2009). Effects of humic substances and quinones at low concentrations on ferrihydrite reduction by *Geobacter metallireducens*. *Environ. Sci. Technol.* 43 5679–5685. 10.1021/es803647r 19731662

[B80] Wu Y. Luo X. Qin B. Li F. Haggblom M. M. Liu T. (2020). Enhanced current production by exogenous electron mediators via synergy of promoting biofilm formation and the electron shuttling process. *Environ. Sci. Technol.* 54 7217–7225. 10.1021/acs.est.0c00141 32352288

[B81] Zachara J. M. Kukkadapu R. K. Fredrickson J. K. Gorby Y. A. Smith S. C. (2002). Biomineralization of poorly crystalline Fe (III) oxides by dissimilatory metal reducing bacteria (DMRB). *Geomicrobiol. J.* 19 179–207. 10.1080/01490450252864271

[B82] Zhang C. Ge Y. Yao H. Chen X. Hu M. (2012). Iron oxidation-reduction and its impacts on cadmium bioavailability in paddy soils: a review. *Front. Environ. Sci. Eng.* 6 509–517. 10.1007/s11783-012-0394-y

[B83] Zhu Y. Wang H. Li X. Hu C. Yang M. Qu J. (2014). Characterization of biofilm and corrosion of cast iron pipes in drinking water distribution system with UV/Cl2 disinfection. *Water Res.* 60 174–181. 10.1016/j.watres.2014.04.035 24859195

[B84] Zhuang K. Izallalen M. Mouser P. Richter H. Risso C. Mahadevan R. (2011). Genome-scale dynamic modeling of the competition between *Rhodoferax* and *Geobacter* in anoxic subsurface environments. *ISME J.* 5 305–316. 10.1038/ismej.2010.117 20668487 PMC3105697

[B85] Ziemke F. Höfle M. G. Lalucat J. Rossellö-Mora R. (1998). Reclassification of *Shewanella putrefaciens* Owen’s genomic group II as *Shewanella baltica* sp. nov. *Int. J. Syst. Evol. Microbiol.* 48 179–186. 10.1099/00207713-48-1-179 9542087

