# Supplementary material for: Impact of Fe(III) (Oxyhydr)oxides Mineralogy on Iron Solubilization and Associated Microbial Communities
Source: Front Microbiol. 2020 Nov 20;11:571244. doi: 10.3389/fmicb.2020.571244 (PMC7715016; doi:10.3389/fmicb.2020.571244)
Supplement: Supplementary Figure 1 — Design and initial compositions of the batch experiments. [file Data_Sheet_1.docx]

Supplementary Material

# Supplementary Figures

## Supplementary Figures


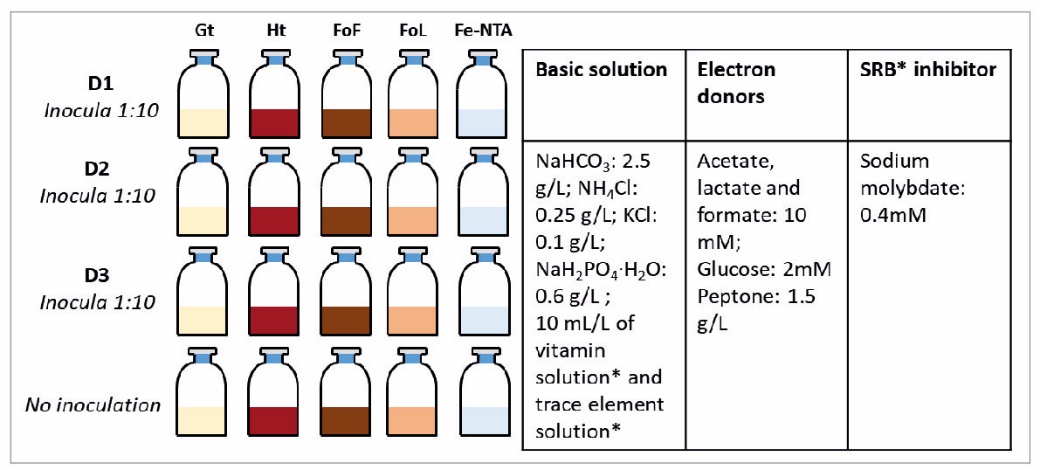


Supplementary Figure S1. Design and initial compositions of the batch experiments

**SRB^1^** : sulfate-reducing bacteria ; 2. **Vitamin solution** (1L): 2 mg biotin, 2 mg folic acid, 10 mg pyridoxine HCL, 5 mg Riboflavin,5 mg Thiamine, 5 mg Nicotinic acid, 5 mg Pantothenic acid, 0.1g B12 vitamin, 5 mg p-aminobenzoic acid, 5 mg thioctic acid ; 3. **Trace element solution** (1L): 1.5 g Trisodium nitrilotriacetic, 0.5 g MgSO_4_, 0.5 g MgSO_4_∙H_2_O, 1 g NaCl, 0.1 g FeSO_4_∙7 H_2_O, 0.1 g CaCl_2_∙2H_2_O, 0.1 g CoCl_2_∙6H_2_O, 0.13 g ZnCl, 0.1 g CuSO_4_ 5H_2_O, 0.1 g AlK(SO_4_)∙12H_2_O, 0.1 g H_3_BO_3_, 0.25 g NaMoO_4_, 0.24 g NiCl∙6H_2_O, 0.25 g Na_2_WO_4_∙2H_2_O

**
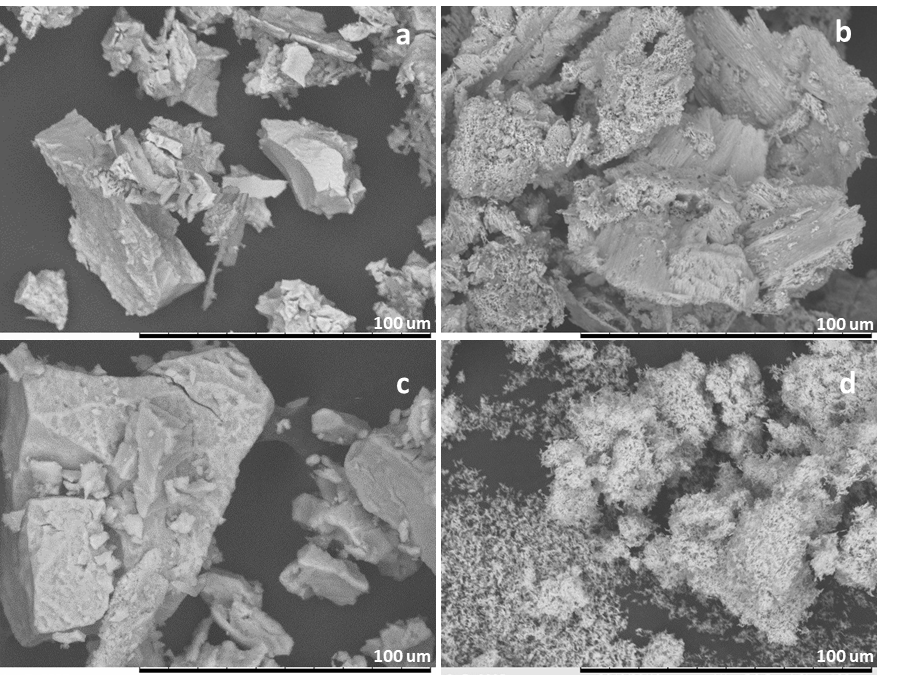
**

**Supplementary Figure S2.** SEM observation on initial Fe (oxyhydr)oxides: Morphology of FoF (a), hematite (b), FoL (c) and goethite (d)

**
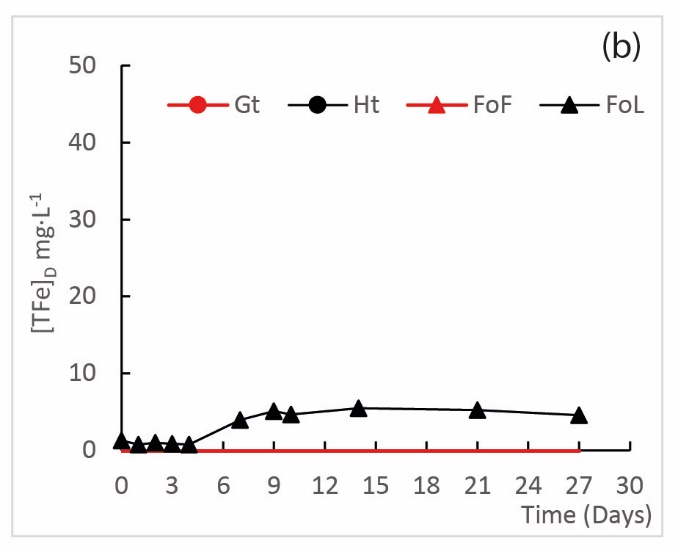
**

**Supplementary Figure S3.** Concentration of Fe(II) and total Fe (FeT) of D1, D2 and D3 incubated on Fe(III)-NTA medium (a), and evolution of the concentration of total Fe during incubation experiments with four abiotic Fe(III) (oxyhydr)oxides (b). Error bars represent the standard deviation of triplicate measurements.

**
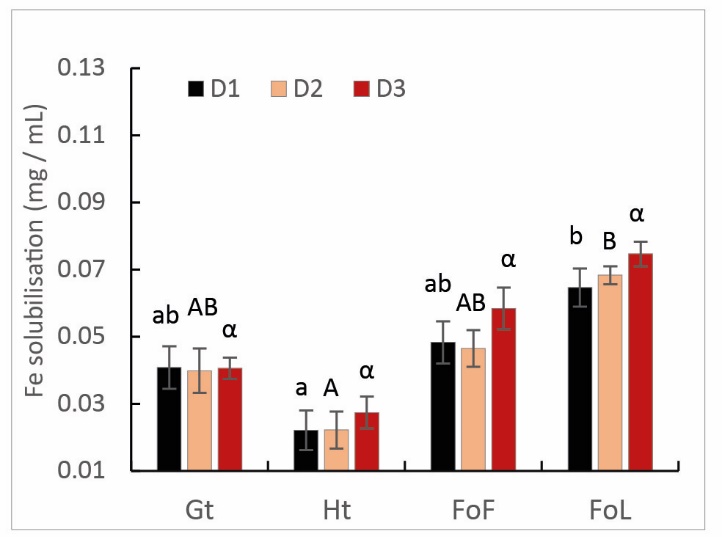
**

**Supplementary Figure S4.** Fe(III) dissolution of Fe oxides: goethite, hematite, FoF and FoL in presence of D1, D2 and D3 inocula. The small letter, capital letter and Greek letter were used for differing significantly (Kruskal-Wallis test at p<0.05) by group of inocula D1, D2 and D3 for different iron oxides. Error bars represent the standard deviation of triplicate measurements.

**
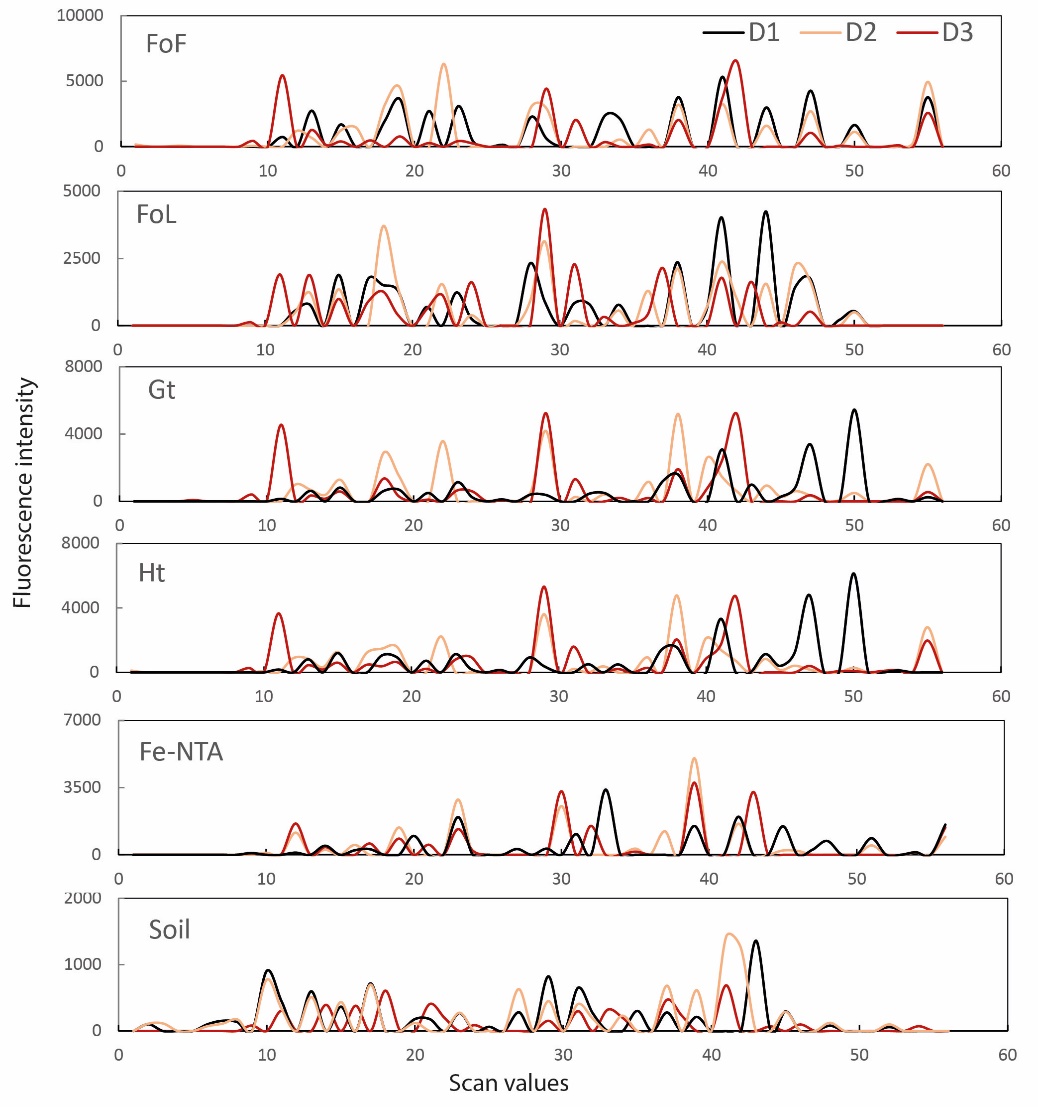
**

**Supplementary Figure S5.** CE-SSCP diversity profiles of the site samples (Soil), the Fe(III)-NTA enrichments (Fe-NTA) and cultures in presence of the four Fe oxides: FoF, FoL, goethite (Gt) and hematite (Ht). D1: soil from river bank; D2: sediment interface, flooded soil; D3: sediment under water. SSCP is a fingerprinting approach, fluorescently labeled blunt-ended PCR products are obtained from the highly variable V3 region of the 16S rRNA (*rrs* gene), denatured at high temperature, and then rapidly cooled to form unique single-strand conformations, which are separated based on their electrophoretic mobility in a polymer-filled capillary (Delbes et al., 2000). The scales are relative to standards. These profiles present high diversity with many peaks, with thus the presence of different bacterial strains.


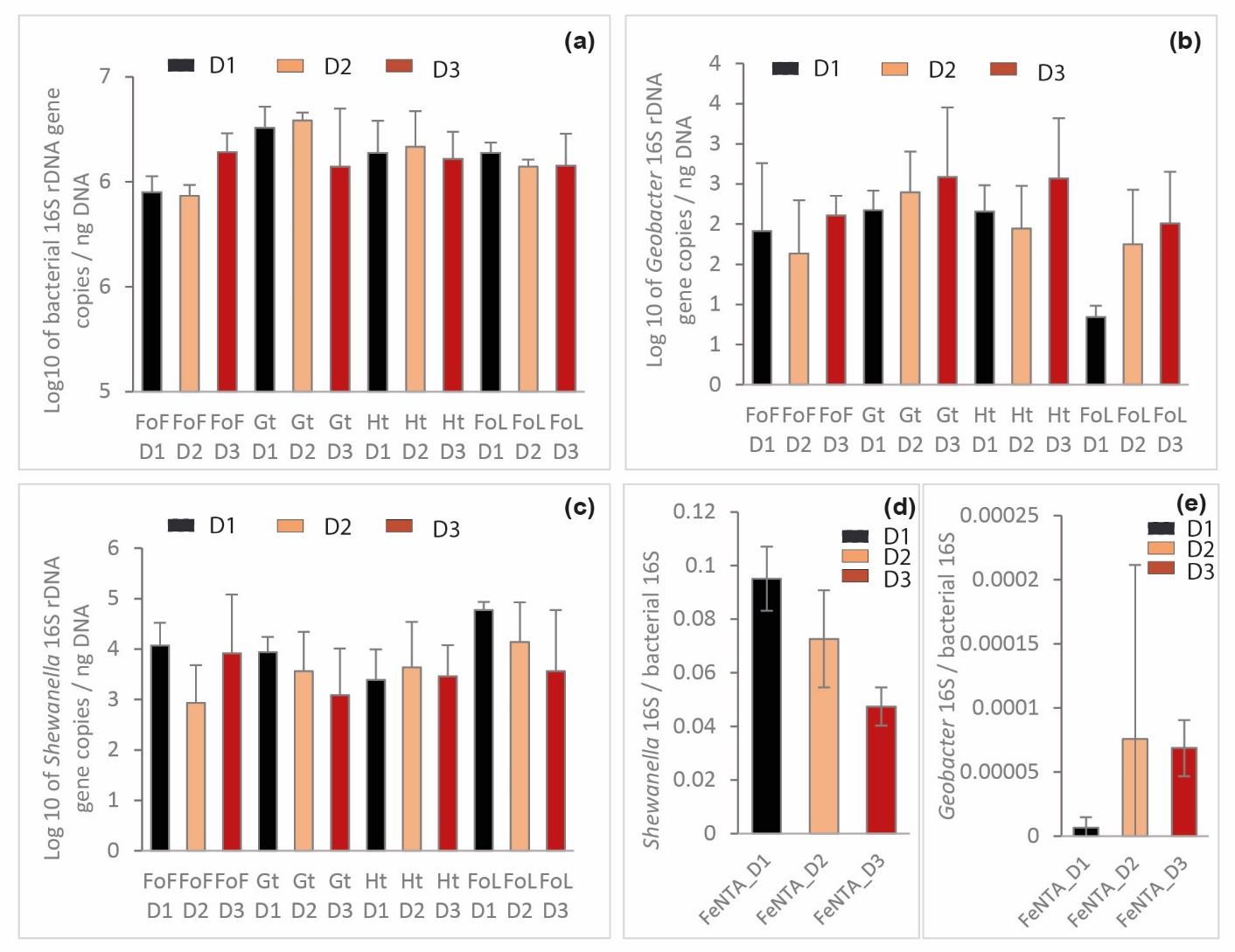


**Supplementary Figure S6.** Parameters linked to bacterial abundance: (**a**) Log10 of bacterial 16S rRNA (*rrs* gene) copies, (**b**) Log 10 of *Geobacter* 16S gene copies, (**c**) Log 10 of *Shewanella* 16S gene copies, for the three site samples D1, D2 and D3; Ratios of *Shewanella* 16S (**d**) and *Geobacter* 16S (**e**) over bacterial 16S rRNA genes copies in Fe(III)-NTA enrichments. Error bars represent the standard deviation of triplicate measurements.

**Supplementary Figure S7.** Principal component analysis (F1 × F2) biplot map generated from iron solubilisation (percentage and rate of iron solubilisation) and ratios of *Shewanella* (*Shewanella* 16S / bacterial 16S) and *Geobacter* (*Geobacter* 16S */* bacterial 16S) in the bacterial community, obtained for the FoF, goethite hematite and FoL incubations with D1, D2 and D3

# Supplementary Tables

## Supplementary Tables

**Supplementary Table S1.** Calculation of the iron oxides concentrations to obtain 20 mM Fe in the incubation experiments

| Minerals | Formula | Fe | Products of Fe(III) oxide |
| --- | --- | --- | --- |
| “Ferrihydrite” FoF | Fe_2_O_3_· 0.5H_2_O | 20 mM | 1.68 g/L |
| Goethite | FeOOH (30-63%: 46%) |  | 3.86 g/L |
| Hematite | Fe_2_O_3_ (97%) |  | 1.65 g/L |
| “Lepidocrocite”  FoL | FeOOH |  | 1.78 g/L |

**Supplementary Table S2.** Initial iron reduction*/solubilisation** rates (mg L^-1^·h^-1^) on incubation experiment with dissolved Fe(III) and solid minerals.

| Inocula/ Minerals | Fe-NTA* | Goethite** | Hematite** | FoF** | FoL** |
| --- | --- | --- | --- | --- | --- |
| D1-1 | 26.17 | 0.07 | 0.03 | 0.09 | 0.47 |
| D1-2 | 29.42 | 0.09 | 0.03 | 0.16 | 0.42 |
| D1-3 | 22.26 | 0.07 | 0.00 | 0.13 | 0.38 |
| D2-1 | 23.51 | 0.07 | 0.04 | 0.16 | 0.42 |
| D2-2 | 24.29 | 0.09 | 0.05 | 0.11 | 0.48 |
| D2-3 | 24.87 | 0.06 | 0.04 | 0.14 | 0.42 |
| D3-1 | 29.21 | 0.08 | NA | NA | 0.46 |
| D3-2 | 23.82 | 0.09 | 0.27 | 0.33 | 0.45 |
| D3-3 | 21.98 | 0.08 | MA | NA | 0.42 |
